# Effortless Willpower? The Integrative Self and Self-Determined Goal Pursuit

**DOI:** 10.3389/fpsyg.2021.653458

**Published:** 2021-03-18

**Authors:** Markus Quirin, Marius Jais, Stefano I. Di Domenico, Julius Kuhl, Richard M. Ryan

**Affiliations:** ^1^School of Management, Technical University of Munich, Munich, Germany; ^2^Department of Psychology, University of Toronto Scarborough, Toronto, ON, Canada; ^3^Department of Psychology, Osnabrueck University, Osnabrück, Germany; ^4^School of Arts and Sciences, Rochester University, Rochester, NY, United States; ^5^Institute for Positive Psychology and Education, Australian Catholic University, Sydney, NSW, Australia

**Keywords:** willpower, volition, integrative self-knowledge, self-determination, personality systems interactions theory

Researchers are increasingly acknowledging a differentiation of two types of “self-management,” namely effortful vs. effortless willpower. For example, recently Ainslie (2020) related effortful willpower to *suppression*, one's ability to gate out imminent urges to maintain a current intention, and effortless willpower to *resolve*, one's ability to motivate oneself to stick to a plan that is considered best on the basis of future incentives and expected temptations.

This differentiation strongly overlaps with differentiations within the behavioral sciences of motivation that have been widely researched, particularly within both Self-Determination Theory [SDT; e.g., Ryan and Deci (2000, 2018)] and Personality Systems Interactions (PSI) Theory [e.g., Kuhl (2000) and Kuhl et al. (2015)]. Both of these theoretical perspectives argue that effortless vs. effortful willpower is strongly related to the degree to which individuals have internalized their goal at hand, and are thus autonomous or willingly motivated to act (Ryan and Deci, 2018). PSI theory construes such volitional motivation as being reliant on a specific neurocognitive network which may be called the *integrative self* (Kuhl et al., 2015). Moreover, the notion that this network can be activated or inhibited by different conditions provides explanations for dynamic switches between effortless vs. effortful willpower over time as well as individual differences thereof. Research within SDT has similarly sought to understand the neurocognitive substrates of volitional motivation and existing studies suggest some points of overlap with PSI theory (Ryan and Deci, 2018).

According to SDT [e.g., Ryan and Deci (2000, 2018)], goal internalization spans a continuum from external regulation (externally pressured or seduced goals), to introjection (goals driven by internal pressures; e.g., feelings of guilt or shame), to identification (personal importance ascribed to goal-related outcomes), up to integration (goals that are congruent with abiding values). The more a goal is internalized, the more self-determined or volitional the individual feels during goal pursuit. There is much evidence showing that higher levels of self-determination are associated with more sustained behavior and optimal performance, including less defensiveness and a lower necessity for suppressive willpower (Ryan and Deci, 2018). However, when goals are regulated externally or through introjections, effortful suppression is more typical. For example, Legault et al. (2009) found that individuals who were more self-determined in their control of prejudice automatically inhibited stereotype application following both a racial prime and previous exertions of self-control.

The neurocognitive mechanisms underlying self-determined motivation have been examined from the perspectives of both PSI and SDT [e.g., Ryan (2017), Quirin et al. (2019), and Koole et al. (2019)]. Specifically, within PSI it has been argued that the different levels of internalization differentially capitalize on the operating of different neuropsychological personality systems that overlap with large-scale cognitive brain networks (Quirin et al., 2019, 2020). Generally, the level of internalization has been linked to the degree to which goals are neurally connected with core representations of the integrative self, an extended parallel-processing network of integrated, personally relevant experiences, goals, values, and preferences. There is evidence that such a system is strongly (yet not exclusively) supported by the ventromedial prefrontal cortex [Kuhl et al., 2015, 2020; Quirin et al., 2019; see also; Northoff et al. (2006), D'Argembeau (2013), and Di Domenico and Ryan (2017)]. This notion is consistent with Ainslie's citations of neuroscientific studies suggesting that effortless willpower relies on the ventromedial (and orbital) prefrontal cortex but that effortful willpower relies on the dorsolateral prefrontal cortex (p. 37).

Rather than a mere passive storage of interconnected experiences, PSI argues that the integrative self is associated with a number of psychological functions that are relevant for effortless willpower: self-positivity, implicit processing, self-decision, self-relaxation, and self-motivation [e.g., Kuhl et al. (2015) and Quirin et al. (2019)]. Self-positivity as one core feature of the integrative self refers to a bias toward a positive evaluation of internalized and self-relevant goals and values. PSI highlights that the stronger a goal is internalized and connected to the self, the stronger the positivity bias, which renders a positive revaluation and resolve more likely. This view is compatible with Ainslie's suggestion that resolve refers to managing motivation to maintain a best plan in the face of expected rewards and temptations—a process that inevitably involves reoccurring revaluations. Maintaining a motivational dominance over time thus requires positive revaluations of that plan, which is facilitated by a greater internalization of goals.

Moreover, the integrative self is considered to operate at an implicit [e.g., Koole and Jostmann (2004) and Quirin et al. (2011)] rather than an explicit level (hence also called the *implicit self*). This is compatible with Ainslie's conceptualization of resolve, which is strongly based on learned habits that do not require conscious awareness and concomitant deliberate decisions against a temptation. Resolve as a process of constant revaluations also entails making difficult decisions in favor of long-term goals and against present tempting alternatives.

*Self-decision* (or “self-determined decision”) is another core function of the self that supports difficult decision making by providing a holistic overview of advantageous, utile options, including remote indicators of how an option relates to personal values, needs, competences, and other self-aspects. Therefore, self-decision is based on the current capability to sense the degree to which a goal preference is self-congruent or based on a self-incongruent, momentary temptation that may need to be effortfully suppressed.

The type of willpower (self-management) that engages the integrative self along with its motivating features has been referred to as *self-regulation* (i.e., “regulated by the self;” Kuhl, 2000), which largely corresponds with the notion of effortless willpower. Two types of self-regulation can be distinguished. *Self-relaxation* refers to self-regulated dampening of negative affect (i.e., supported by the integrative self), whereas *self-motivation* (self-determined motivation) refers to the self-regulated upregulation of positive affect required to enact one's intentions; and individuals differ in either dimension (Kuhl and Fuhrmann, 1998).

Self-regulation may be contrasted with *self-control* (or “self-discipline”), which uses deliberate, effortful suppression of potentially distracting goals, needs, or impulses, and thus excludes rather than involves the integrative self in the context of goal pursuit.

Research in SDT similarly distinguishes self-regulated vs. self-controlling motivations, and argues that more integrated, autonomous motivation requires access to processes relevant not only for detecting motivational conflicts, but also to the self-relevant information needed to resolve them. For example, autonomous motivation is associated with greater neurophysiological reactivity to errors during the performance of Go/No-Go and Stroop tasks (Legault and Inzlicht, 2013). Moreover, during personally relevant decision making, people who experience more proximal support for self-determination evidence longer reaction times and greater conflict-related activity in both the anterior cingulate cortex (ACC) and medial prefrontal cortex (MPFC) when forced to choose between closely preferred options (Di Domenico et al., 2013, 2016). This research suggests that both the affective evaluative systems for monitoring motivational conflicts (ACC) and the self-knowledge executive systems for resolving motivational conflicts (MPFC) are more readily recruited in people who are more self-determined. Of course, maintaining motivation for a long-term goal (i.e., resolve) also means coping with setbacks, self-doubt, and rumination about tempting goal alternatives. SDT research has repeatedly shown greater sustained attention and effort to tasks that are self-regulated, that is, performed autonomously (Ryan and Deci, 2018).

Lastly, within both PSI and SDT, goals with a high level of internalization or strong connectedness with the integrative self provide the energy to stick to a goal even in the face of tempting goal alternatives by enabling the individual to experience positive emotions derived from the congruence with many interconnected and positively biased self-aspects. As such, self-determined motivation is inherently linked to more effortless willpower as it renders effortful shielding of a chosen goal from competing alternatives (i.e., effortful suppression) superfluous. The fact that one is not “fighting against oneself” is evidenced by the greater vitality and lessened depletion shown by people enacting autonomous vs. less internalized goals (Moller et al., 2006; Kazén et al., 2015).

According to PSI theory, the integrative self can be relatively activated or inhibited. Activation of the integrative self enables the manifest expression of the functions described above, whereas inhibition keeps them silent—a mechanism that strongly contributes to the variability in behavior and experience. In PSI, activation of the neuropsychological network supporting the integrative self creates *self-access* (Quirin and Kuhl, 2018), and studies have demonstrated that self-access can be inhibited by conditions such as negative affect or stress (Quirin et al., 2009), particularly in individuals with low emotion regulation abilities (Kuhl and Kazén, 1994; Baumann and Kuhl, 2003; Baumann et al., 2005). Similarly, experiments and experience sampling studies guided by SDT show that support in the fulfillment of psychological needs facilitate greater self-access (e.g., Weinstein et al., 2013).

It is also important to mention that the distinction between effortful vs. effortless behavior involves two considerations: the effort needed to stay “on task” and the effort dedicated to performing the task itself. Data from both PSI and SDT show that people tend to put more effort into tasks they value and autonomously do; at the same time, working on tasks volitionally is less draining and depleting than doing tasks for introjected or externally regulated reasons. That is, doing what one truly values can take effort, but it is even harder to “make oneself” or “keep oneself” doing what one does not stand behind. Ainslie's effortful willpower, therefore, seems particularly apt for characterizing the demands of staying “on task” when pursuing goals that are not well-internalized.

Clearly, the differentiation between the two types of willpower offered by Ainslie (2020) partly overlaps with evidence from both the PSI and SDT perspectives. The present commentary also puts the “jingle-jangle” problem of willpower research into sharper relief. Sometimes, researchers use the same term to describe different constructs (“jingle”); other times, they use different terms to describe the same constructs (“jangle”). Without a clear theory for drawing distinctions, neglecting to distinguish between effortful and effortless willpower (i.e., self-control vs. self-regulation) may more likely. To this point, Milyavskaya et al. (2019) pointed out that whereas some studies have found dispositional “self-control” to be positively related to successful exertions of “self-control” (e.g., DeWall et al., 2007), others have reported opposite results (e.g., Imhoff et al., 2014). This apparent paradox may partly stem from terminological confusions about the meaning of willpower. If some measures of dispositional self-control actually assess effortful willpower, we would expect them to be associated with more situational akrasia.

Similarly, the present work also alludes to the problem of not distinguishing (“jingling”) between a *lack* of impulsivity and high self-control (Moffitt et al., 2011). PSI research suggests that the integrative self becomes activated in response to threat (fostering self-relaxation) or frustration/amotivation (fostering self-motivation), but that individuals differ in these dynamics (Baumann et al., 2017). Accordingly, high self-regulation competencies are particularly important for individuals with increased sensitivities to threat or frustration. Milyavskaya et al. (2019) pointed out that the terms “self-control” and “self-regulation” are often used interchangeably. Herein we have attempted to underscore the importance of distinguishing these constructs by linking “self-regulation” to less effortful willpower, which in both PSI and SDT perspectives, is seen as being supported by the activation of the integrative self.

In sum, both PSI theory and SDT can strongly contribute to explaining the dynamics of effortful vs. effortless willpower as they unfold over time and situations, and according to individual differences. We believe that the conceptual distinction between effortful and effortless willpower [e.g., Ainslie (2020)] on the basis of an integrative view from SDT and PSI theory represents a fertile ground for future research.

## Author Contributions

MQ conceived the outline for this commentary. MQ (in charge) and MJ (contributorily) wrote the first draft of the manuscript. Finally, SD, RR, and JK amended and revised it. All authors contributed to the article and approved the submitted version.

## Conflict of Interest

The authors declare that the research was conducted in the absence of any commercial or financial relationships that could be construed as a potential conflict of interest.
