# Corrigendum: Effortless Willpower? The Integrative Self and Self-Determined Goal Pursuit

**DOI:** 10.3389/fpsyg.2021.684433

**Published:** 2021-05-05

**Authors:** Markus Quirin, Marius Jais, Stefano I. Di Domenico, Julius Kuhl, Richard M. Ryan

**Affiliations:** ^1^School of Management, Technical University of Munich, Munich, Germany; ^2^Department of Psychology, University of Toronto Scarborough, Toronto, ON, Canada; ^3^Department of Psychology, Osnabrueck University, Osnabrück, Germany; ^4^School of Arts and Sciences, Rochester University, Rochester, NY, United States; ^5^Institute for Positive Psychology and Education, Australian Catholic University, Sydney, NSW, Australia

**Keywords:** willpower, volition, integrative self-knowledge, self-determination, personality systems interactions theory

In the original article, there is a redundant heading at the start of the text that reads:

*EFFORTLESS WILLPOWER? THE INTEGRATIVE SELF AS A NEUROPSYCHOLOGICAL SYSTEM FACILITATING EFFORTLESS WILLPOWER THROUGH SELF-DETERMINED REGULATION*

This heading should be removed from the article.

The authors apologize for this error and state that this does not change the scientific conclusions of the article in any way. The original article has been updated.

